# Mitral valve nonbacterial thrombotic endocarditis: a rare multi-surgery-tolerant survivor of Trousseau’s syndrome

**DOI:** 10.1186/s40792-018-0513-5

**Published:** 2018-08-29

**Authors:** Yoshiharu Soga, Kaoru Taira, Akira Sugimoto, Manabu Kurosawa, Hiromasa Kira, Takamitsu Su, Kazuhiko Doi, Akira Nakano, Yoshihiro Himura

**Affiliations:** 1 Department of Cardiovascular Surgery, Nagahama City Hospital, Nagahama, Japan; 2 Department of Surgery, Nagahama City Hospital, Nagahama, Japan; 3 Department of Cardiology, Hikone Municipal Hospital, 1882 Yasaka-cho, Hikone, Shiga 522-8539 Japan; 4 Department of Pathology, Nagahama City Hospital, 313 Oh-inui-cho, Nagahama, Shiga 526-8580 Japan

**Keywords:** Trousseau’s syndrome, Nonbacterial thrombotic endocarditis, Direct oral anticoagulant, Heparin, Cardiac surgery

## Abstract

**Background:**

Few previous reports have documented cases of nonbacterial thrombotic endocarditis associated with Trousseau’s syndrome for which surgery proved possible for both the primary tumor and the cardiac lesion. The effectiveness of direct oral anticoagulants in patients with Trousseau’s syndrome has also received scant attention.

**Case presentation:**

A 69-year-old man with repeated episodes of cerebral infarction was diagnosed as having nonbacterial thrombotic endocarditis after mitral valve replacement surgery. Stroke recurred preoperatively under apixaban administration. A stomach biopsy also identified gastric adenocarcinoma, and gastric surgery was performed on the 40th postoperative day. The patient was discharged from the hospital and has been free of thromboembolism under a regime of subcutaneous heparin self-injection thereafter.

**Conclusions:**

We have reported a rare multi-surgery-tolerant survivor of Trousseau’s syndrome in whom subcutaneous heparin injection was useful for preventing thromboembolic events over a long period.

## Background

Trousseau’s syndrome is defined as unexplained thrombotic events that precede a diagnosis of occult visceral malignancy [1]. Among various etiologies, nonbacterial thrombotic endocarditis (NBTE) has increasingly been recognized as a potentially life-threatening source of thromboembolism [2].

We present an extremely rare case of Trousseau’s syndrome in which the patient had undergone surgery for gastric adenocarcinoma following mitral valve replacement for NBTE. The patient was heparinized and survived for 18 months without recurrence of thromboembolism.

## Case presentation

A 69-year-old male was admitted to a hospital in June 2016 because of right arm asthenia and dysarthria and was diagnosed as having cerebral infarction in the left middle cerebral artery area along with deep vein thrombosis. ECG demonstrated normal sinus rhythm and echocardiography revealed no intra-cardiac thrombus or vegetation. The patient was discharged from the hospital following administration of apixaban.

In August 2016, the patient was readmitted to the hospital because of recurrent right arm asthenia and dysarthria. MRI revealed multiple cerebral infarctions in not only the bilateral cerebral hemispheres but also the cerebellum. Trousseau’s syndrome was suspected at this time. Apixaban administration was stopped and an intravenous drip of heparin was started. Echocardiography revealed mild mitral regurgitation with vegetation on the mitral valve. Although the laboratory data suggested no evidence of infection, ceftriaxone and gentamicin were added as a precaution against infective endocarditis. The patient was then referred to our hospital for surgery.

**Fig. 1:**
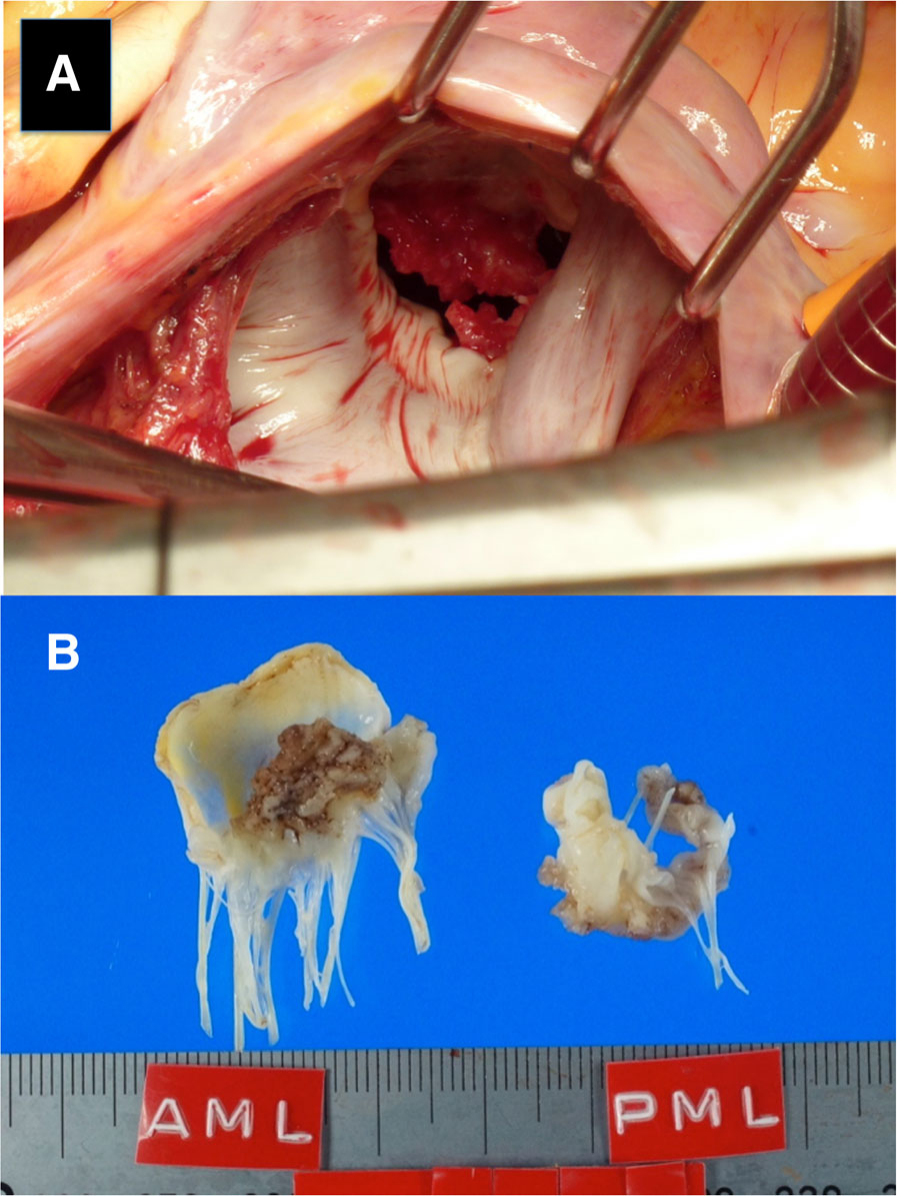
Intraoperative photograph showing the mitral valve and vegetation on the both mitral leaflets (**a**). Representative photographs of the extracted mitral leaflets (**b**)

**Fig. 2:**
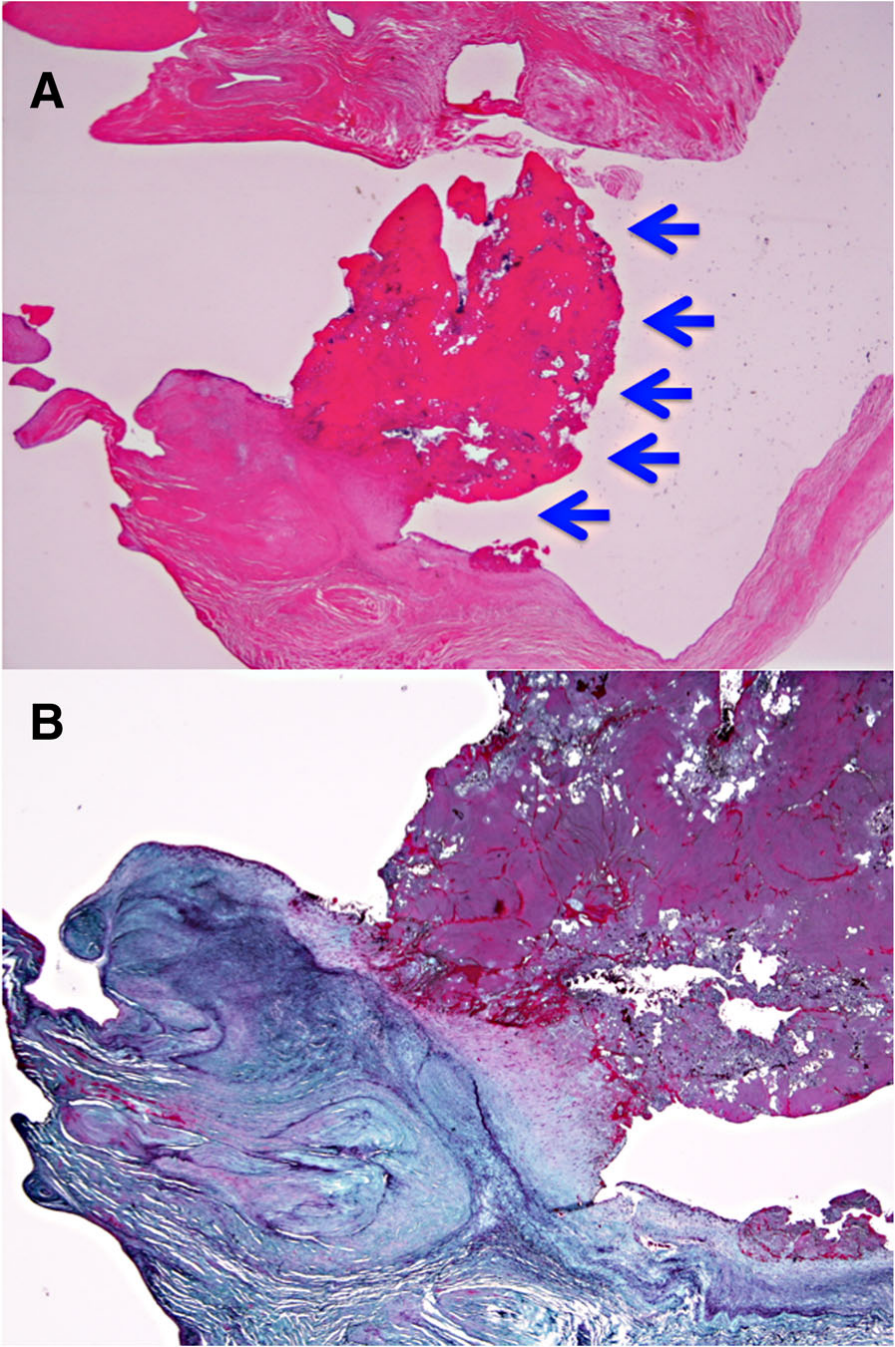
Histological findings of vegetation in the mitral valve. No neutrophil infiltration or bacterial agglomeration is evident. Arrows indicate vegetation (hematoxylin and eosin stain, magnification × 10) (**a**). No valve tissue damage was seen (Elastica Masson stain, magnification × 20) (**b**)

A CT scan revealed a left renal infarction and multiple swollen lymph nodes around both the abdominal aorta and stomach with antral hypertrophy, suggesting an advanced gastric cancer or lymphoma. As the vegetation showed no change despite the heparin and antibiotics therapy, cardiac surgery was performed on day 5 after referral. Extracorporeal circulation was instituted employing aortic and bicaval cannulation. After aortic cross-clamping, the mitral valve was exposed via a left atriotomy. Both mitral leaflets had vegetation on the surface, and major vegetation 15 mm in width was evident on the anterior leaflet (Fig. 1). These were resected in their entirety and replaced with a 25-mm Epic bioprosthesis (Abbott). Continuous intravenous heparin administration was resumed on the following day, aiming for an activated partial thromboplastin time of between 40 and 50 s. Histologic analysis revealed that the vegetations were thrombi covered with vascular endothelium and that the mitral leaflet tissue was not damaged (Fig. 2). On the basis of these findings, the patient was diagnosed as having NBTE.

An endoscopic stomach biopsy was performed on the seventh postoperative day, and histologic analysis revealed non-solid poorly differentiated adenocarcinoma with components of signet-ring cell carcinoma and moderately differentiated tubular adenocarcinoma. The patient was definitively diagnosed as having Trousseau’s syndrome and, subsequently, transferred to the department of surgery. A Billroth I distal gastrectomy was performed, and a continuous intravenous heparin drip was employed during the operation. Histologic analysis revealed poorly differentiated adenocarcinoma with a component of moderately differentiated tubular adenocarcinoma and metastatic tumor cells in the dissected lymph nodes (T4aN3bM0; stage IIIb). Further histologic analysis using alcian blue staining confirmed the presence of mucin in the tumor.

Subcutaneous heparin injection was introduced on day 8 after the gastric surgery, and the patient was discharged from our hospital in October after acquisition of the self-injection technique. During this long hospitalization, no thromboembolic events were observed. Chemotherapy was started in November. The patient has survived for 18 months after the diagnosis of Trousseau’s syndrome without any recurrence of thromboembolism.

## Discussion

Trousseau’s syndrome often occurs in patients with carcinoma of the pancreas, ovary, and lung. As mucin-producing adenocarcinoma from the gut or lung is commonly associated with NBTE [3, 4], NBTE can be one of the important primary episodes leading to a diagnosis of Trousseau’s syndrome.

The pathogenesis of NBTE is not fully understood, but the lesions classically occur in areas of high flow on valve leaflets. Blood flow may therefore contribute to the location of vegetation [3]. It has been advocated that the pathogenesis may involve tissue factors and tumor-associated cysteine protease activity, tumor hypoxia, carcinoma mucin activity associated with platelet aggregation, and oncogene activation related to hypercoagulability [1]. Of these, production of mucin by carcinoma is thought to play an important role in the onset of Trousseau’s syndrome [5, 6].

Histological examination of the resected valve remains the gold standard for diagnosis of NBTE. Culture-negative endocarditis can only be excluded histologically when there is a lack of acute valvular inflammation and absence of fungi or bacteria [7].

According to the latest European guidelines for NBTE, surgical intervention, valve debridement, and/or reconstruction are often not recommended unless the patient presents with recurrent thromboembolism despite well-controlled anticoagulation. Other indications for valve surgery are the same as for infective endocarditis. In the context of cancer, a multidisciplinary approach is recommended [8]. Valve surgery was indicated for the present patient because the size of the vegetation had not changed despite anticoagulation.

There have been few reported cases of NBTE with Trousseau’s syndrome in which cardiac surgery has been possible [9]. One major reason may be that most tumors are at an advanced stage at the time of detection. Indeed, Cestari et al. investigated patients with Trousseau’s syndrome in whom stroke had been the primary event and reported that the median survival was 4.5 months from the diagnosis of stroke; 25% of the patients died within 30 days [10].

According to the above guidelines, if there is no contraindication, NBTE patients should be anticoagulated with unfractionated or low-molecular-weight heparin or warfarin, although there is little evidence to support this strategy [8]. Some investigators do not recommend warfarin usage because of the presence of non-vitamin K-dependent agents that may induce thrombotic coagulopathy [11]. Few studies have investigated the effectiveness of direct oral anticoagulants in patients with Trousseau’s syndrome. Dabigatran has been reported to be ineffective for prevention of recurrent ischemic stroke [12]. In the present patient, stroke recurred under apixaban administration preoperatively. Accordingly, we chose heparin, and no recurrent thromboembolic events were subsequently observed in the patient.

## Conclusion

We have reported a rare multi-surgery-tolerant survivor of Trousseau’s syndrome in whom **s**ubcutaneous heparin injection was useful for preventing thromboembolic events over a long period.
